# Methanogenic recovery under organic overload using starfish-derived powder

**DOI:** 10.3389/fmicb.2026.1853600

**Published:** 2026-06-17

**Authors:** Hyeongyu An, Juhee Shin, Ilho Bae, Minjae Kim, Jin Mi Triolo, Seung Gu Shin

**Affiliations:** 1 Department of Energy System Engineering, Gyeongsang National University, Jinju, Gyeongnam, Republic of Korea; 2 Future Convergence Technology Research Institute, Gyeongsang National University, Jinju, Gyeongnam, Republic of Korea; 3 Department of Civil Engineering, University of Kentucky, Lexington, KY, United States

**Keywords:** anaerobic digestion, calcium carbonate, ossicles, pH buffering, starfish, triphasic

## Abstract

**Introduction:**

Starfish overpopulation causes ecological and economic problems, and the collected biomass is typically discarded by incineration or landfilling. Given its high CaCO_3_ content and biodegradable fraction, starfish biomass could be valorized as a multifunctional additive in anaerobic digestion (AD). This study evaluated the buffering and recovery effects of starfish powder (SF) under starch-induced overloading.

**Methods:**

Starfish (*Asterina pectinifera* and *Asterias amurensis*) were dried, pulverized (<1.7 mm), and characterized for physicochemical properties and theoretical biochemical methane potential (TBMP). Long-term (257 days) batch assays compared starch overload (30 g volatile solids (VS)/L) alone, CaCO_3_ supplementation, and SF addition at equivalent or higher CaCO_3_ doses.

**Results:**

As a sole substrate, SF yielded 490 mL CH_4_/g VS (59–73% of TBMP). Under starch overloading, CaCO_3_ supplementation failed to restore activity (≤5.4% of expected yield), whereas SF enabled recovery with triphasic methane production, reaching 60–78% of the expected yield. Microscopy indicated more open pores with microbial colonization in the SF trials, and 16S rRNA sequencing analysis indicated a shift in fermentative bacterial communities accompanied by enrichment of hydrogenotrophic methanogens.

**Discussion:**

These findings suggest that SF promotes recovery through combined physicochemical buffering and microbial restructuring, serving as a slow-acting alkalinity source in overloaded digesters where long-term stability is prioritized.

## 1 Introduction

Starfish overpopulation has been reported globally, causing ecological disruption and economic losses in aquaculture (Qu et al., 2024). Outbreaks of species such as *Acanthaster planci*, *Asterias amurensis*, and *Asterias rubens* have damaged coral reefs and shellfish farms, driven by predator decline, seawater warming, and nutrient enrichment that enhance larval survival and recruitment (Ross et al., 2003; Kroon et al., 2021; Liu et al., 2021). In Korea, *A. amurensis* poses a major threat to shellfish and abalone farming. Most collected biomass is incinerated or landfilled, incurring high costs and environmental burdens, despite the large volumes available annually—indicating substantial potential for resource recovery (Qu et al., 2024).

In anaerobic digestion (AD), one of the most common causes of process instability is rapid pH reduction due to organic overloading, which suppresses methanogenic activity (Veeken et al., 2000). The pH control strategies can be broadly categorized into: (i) direct addition of hydroxide-based reagents such as NaOH and Ca(OH)_2_, which raise pH immediately but can induce localized osmotic/ionic shock to microbial communities and often require frequent reapplication; and (ii) supplementation of buffering ions, which stabilize pH through reversible acid–base equilibria (Chen et al., 2008; Zhang et al., 2015). Buffering ions in AD include bicarbonate and other weak acid–base pairs (Lin et al., 2013). Among these, HCO_3_^–^ is the primary buffer in methanogenic digesters, maintaining pH near the optimal range by neutralizing protons generated during volatile fatty acid (VFA) accumulation (Batstone et al., 2002; Appels et al., 2011). However, due to pH-dependent carbonate speciation, when the bulk pH drops below ∼6.5, HCO_3_^–^ shifts toward dissolved CO_2_ and readily degasses from the liquid, thereby diminishing buffering capacity in open systems (Photiou and Vyrides, 2022).

Accordingly, a range of approaches has been investigated to mitigate pH decline during anaerobic digestion, including controlled organic loading, co-digestion with alkaline substrates, addition of chemical buffers or alkaline wastes, and supplementation of solid materials such as biochar, minerals, or metal oxides to enhance buffering capacity and microbial stability (Wang et al., 2021).

While these strategies can delay acidification or accelerate recovery, many rely on rapid chemical intervention or continuous dosing, and their effectiveness often decreases under severe or prolonged overloading conditions (Veeken et al., 2000).

Against this background, starfish biomass offers unique multifunctional potential for AD application. First, its organic fraction can serve as a co-substrate, contributing biodegradable material and additional methane yield (Ferdeş et al., 2023). Second, its ossicles are composed predominantly of CaCO_3_ (Blowes et al., 2017; Yang et al., 2022) which can act as an alkalinity enhancer by releasing HCO_3_^–^ to counteract acidification (Notodarmojo et al., 2021; Photiou and Vyrides, 2022). Third, its porous ossicle microstructure can facilitate microbial colonization, similar to other inorganic carriers in AD systems (Blowes et al., 2017; Notodarmojo et al., 2021; Photiou and Vyrides, 2022). Thus, starfish could simultaneously provide organic substrate, chemical buffering capacity, and structural support for microbial communities.

In this study, the potential of starfish as a multifunctional additive in anaerobic digestion was evaluated under starch-induced overloading. Methane yield and buffering performance were assessed, and recovery kinetics were characterized by fitting cumulative methane production to the modified Gompertz model to determine the lag phase (λ), methane potential (*B*_0_), and maximum methane production rate (*R*_*m*_). Two treatments were compared: CaCO_3_-only addition and starfish addition at an equivalent CaCO_3_ dose, enabling direct evaluation of purely chemical effects versus the combined chemical–organic–structural effects of starfish.

This study reframes starfish as an underutilized marine biomass functioning as a three-dimensional CaCO_3_ source that mitigates the buffering limitations of conventional CaCO_3_ under acidic anaerobic digestion conditions.

## 2 Materials and methods

### 2.1 Materials

Dried starfish specimens were obtained from a local collection and processing program in Geoje City, South Korea, which was aimed at managing excess starfish generated during fishing activities. The samples consisted of *Asterina pectinifera* and *Asterias amurensis* in a number ratio of 5:1. The dried starfish were mixed with distilled water at a 1:1 (*w*/*w*) ratio and pulverized (<1.7 mm) using a grinder, followed by drying at 105°C for 24 h.

### 2.2 Characterization

Particle size distribution was determined using sieves with mesh sizes of 1.7, 0.85, and 0.15 mm. Total solids (TS) content was measured according to the Standard Methods of the American Public Health Association. The higher heating value (HHV) was measured according to ASTM D5865 by combusting ∼1 g of sample under 3 atm oxygen in a 5E-C5500 calorimeter (Changsha Kaiyuan Instruments, China). The elemental composition, including carbon (C), hydrogen (H), nitrogen (N), and sulfur (S), was analyzed using an elemental analyzer (Vario Macro Cube, Elementar, Germany).

### 2.3 Theoretical biochemical methane potential estimation

The empirical formula of starfish powder was determined from its elemental composition (C, H, O, N, S). The theoretical biochemical methane potential (TBMP) at standard temperature and pressure (0°C, 1 atm) was calculated using the Buswell–Boyle stoichiometric equations (Equations 1 and 2) for an organic compound C_a_H_b_O_C_N_d_S_e_.


$$C_{a}\,H_{b}\,O_{C}\,N_{d}\,S_{e}+\left(\frac{8\,a-2\,b-4\,c+6\,d+4\,e}{8}\right)\,H_{2}\,O$$ (1)

$$\to \left(\frac{4\,a+b-2\,c-3\,d-2\,e}{8}\right)\,{\mathrm{CH}}_{4}+\left(\frac{4\,a-b+2\,c+3\,d+2\,e}{8}\right)$$

$${\mathrm{CO}}_{2}+{\mathrm{dNH}}_{3}+{\mathrm{eH}}_{2}\,S$$

$$\mathrm{TBMP}\,\left(\mathrm{mL}\,{\mathrm{CH}}_{4}/g\,\mathrm{VS}\right)=2,800\times \left\{\frac{4\,a+b-2\,c-3\,d-2\,e}{12\,a+b+16\,c+14\,d+32\,e}\right\}$$ (2)

### 2.4 Batch experiments

The inoculum was collected from a mesophilic anaerobic digester at Jinju Wastewater Treatment Plant (Jinju, South Korea) and was subjected to a 2-week starvation period prior to use. Its pH, total solids (TS), and volatile solids (VS) were 7.36, 20.8, and 14.0 g/L, respectively.

Batch experiments were conducted in 110 mL serum bottles at 35 ± 1°C in triplicate. Each reactor was loaded with 36 mL of inoculum and amended with substrates and deionized water to a final working volume of 40 mL.

For evaluation of the methane yield of starfish powder (SF), the treatments included an inoculum-only control, a positive control with microcrystalline cellulose (CEL), and an SF-only condition at 15 g VS/L, corresponding to 50 g fixed solids (FS)/L.

For starch-based overloading experiments, starch (wheat flour; Daehan Flour Co., South Korea) was used as the primary substrate, while calcium carbonate (CaCO_3_; >98%; Daejung Chemicals, South Korea) and SF were applied as additives. All materials were supplied in powder form. Organic overloading was induced by starch addition at a concentration of 30 g VS/L. CaCO_3_ was supplemented at 5, 25, or 50 g/L. SF was supplied at 5, 25, or 50 g FS/L, corresponding to 1.5, 7.5, and 15 g VS/L, respectively.

Before operation, all reactors were purged with N_2_ gas to establish anaerobic conditions. Biogas volume was measured daily at first, then at 2–7 day intervals. For each measurement, headspace pressure was checked with a digital manometer (LEO2, Keller, Switzerland) and released with a syringe to ambient pressure, with sampling time and volume recorded. Reactors were operated under static conditions and were gently shaken only on measurement days after pressure release. CH_4_ and CO_2_ contents were determined using a gas chromatograph (GC-2030, Shimadzu, Japan) equipped with a gas separation column (CP7485, Agilent, United States) and a thermal conductivity detector. Gas composition was first measured on day 6, then at ≤2-week intervals to day 52, every 3–4 weeks to day 100, and about every 2 months thereafter.

At the end of the experiment, digestate pH, volatile fatty acids (acetic, propanoic, isobutyric, butyric, isovaleric, valeric, isocaproic, and caproic acids), and ethanol were analyzed using a gas chromatograph (GC-2030, Shimadzu, Japan) equipped with an organic acid column (113-3133, Agilent, United States) and a flame ionization detector. End-point alkalinity was also measured using the same digestate samples by potentiometric acid titration with an automatic titrator (TitroLine^®^ 5000, SI Analytics, Germany). The samples were titrated to endpoint pH values of 4.3 and 3.8, and the results were expressed as mg CaCO_3_/L. SF before and after digestion was examined by field-emission scanning electron microscopy (FE-SEM; MIRA3, TESCAN, Czech Republic) following fixation with 2.5% glutaraldehyde, post-fixation with 1% osmium tetroxide in 0.1 M phosphate buffer (pH 7.4), and dehydration through a graded ethanol series.

### 2.5 Modeling

Cumulative methane production in the SF-supplemented treatments that resumed methane production was fitted using a tri-phasic modified Gompertz model:


$$B\,\left(t\right)=\sum_{i=1}^{3}B_{0,i}\,\exp\,\left\{-\exp\,\left[\frac{R_{m,i}\,e}{B_{0,i}}\,\left(\lambda_{i}-t\right)+1\right]\right\}$$

where *B*(*t*) is the cumulative methane production at time *t*, *B*_0,*i*_ is the methane production potential of phase *i*, *R*_*m,i*_ is the maximum methane production rate of phase *i*, and λ_*i*_ is the absolute lag time of phase *i* from day 0. For the single-substrate methane yield tests using SF and cellulose (CEL), the equation was applied with *n* = 1 to estimate *B_0_*, *R_m_*, and λ. For the SF-supplemented overload treatments that showed triphasic methane recovery, the equation was applied with *n* = 3 as a tri-Gompertz model. The units of *B*(*t*) and *B*_0,*i*_ were expressed as mL CH_4_/g VS for the single-substrate methane yield tests and as mL CH_4_/bottle for the starch-overload recovery tests. The units of *R*_*m,i*_ were expressed accordingly as mL CH_4_/g VS/day or mL CH_4_/day.

For the single-phase SF and CEL methane yield tests, model fitting was performed using SigmaPlot version 12.0 and R version 4.3.2 with the minpack.lm package. For the tri-Gompertz fitting of the SF-supplemented overload treatments, model fitting was performed at two levels. First, each replicate bottle was fitted individually to obtain bottle-specific fitted curves and kinetic parameters, which are presented in Supplementary Figure 1 and Supplementary Table 1. Second, for Figure 3, treatment-averaged experimental curves were obtained by averaging the three replicate bottles at each sampling time and were then fitted directly using the same model.

For the tri-Gompertz fitting, the entire cumulative methane production curve was fitted simultaneously using a single tri-Gompertz model, without dividing the data into separate phase-wise intervals. Initial parameter values were selected only to facilitate convergence of the nonlinear regression and were allowed to vary during optimization. No fixed time boundaries were imposed between the three Gompertz phases. Nonlinear regression was performed in Python using the least_squares optimization routine from the SciPy package. *B*_0,*i*_ and *R*_*m,i*_ were constrained to positive values, and the lag times were constrained to satisfy λ_1_ < λ_2_ < λ_3_ to maintain the chronological order of methane production phases. Model performance was evaluated using the root mean square error (RMSE) and the coefficient of determination (*R*^2^).

For the triphasic model, the total fitted methane production potential was calculated as the sum of the three phase-specific methane production potentials:


$$B_{0,t\,o\,t\,a\,l}=B_{0,1}+B_{0,2}+B_{0,3}$$

Tri-Gompertz kinetic parameters obtained from individual replicate bottles were compared among the SF-supplemented treatments that resumed methane production. For each treatment, parameter values are reported as mean ± standard deviation (SD, *n* = 3), with the sample standard deviation used to represent replicate-level variability. Given the small replicate number and unequal replicate-level variability among treatments, treatment effects for each parameter were assessed using Welch’s ANOVA, and pairwise differences among treatments were evaluated using Games–Howell *post-hoc* tests. Statistical significance was accepted at *p* < 0.05. Different superscript letters within the same parameter indicate significant differences based on the Games–Howell *post-hoc* tests, whereas treatments sharing at least one letter are not significantly different. The full statistical summary of all fitted kinetic parameters is provided in Supplementary Table 1.

### 2.6 Microbial analysis

At the end of the batch tests, digestate from each replicate was pooled and pelleted for DNA extraction (AccuPrep genomic DNA extraction kit, Bioneer, South Korea). Bacterial and archaeal 16S rRNA amplicon sequencing was performed on the Illumina iSeq 100 (151 × 2 paired-end reads) as previously described (Shin et al., 2021). Sequences were clustered *de novo* into operational taxonomic units (OTUs) at 97% sequence identity using VSEARCH (Rognes et al., 2016). Taxonomic classification was performed using the RDP Classifier (Wang et al., 2007; v2.14), run locally via a Java command-line interface with an 80% confidence cutoff; OTUs below this threshold were labeled as unclassified. Each sample yielded >21,000 valid reads.

## 3 Results and discussion

### 3.1 Characterization of SF

The physicochemical properties of SF are summarized in Table 1. Nearly all (99.4%) SF particles were smaller than 1.7 mm, with 50.2% smaller than 0.15 mm. The TS, VS, and fixed solids (FS) contents were 98.7, 23.1, and 75.6% of the wet weight, respectively, and the calorific value was 1,905 cal/g TS (van der Heide et al., 2018).

**TABLE 1:** Basic characteristics of SF.

Parameter		Value
Total solids (%)		98.7
Volatile solids (%)		23.1
Fixed solids (%)		75.6
Calorific value (cal/g TS)		1,905
Particle Size Distribution (%)	>1.7 mm	0.6
	0.85–1.7 mm	14.1
	0.15–0.85 mm	35.2
	<0.15 mm	50.2

Based on the elemental analysis of C, H, N, and S, the organic C content, oxygen (O) content, and TBMP were calculated assuming CaCO_3_ contents in FS from 50 to 80% (Table 2). Previous studies have reported CaCO_3_ to constitute 49–75% of starfish dried weight (Lebrato et al., 2010). In addition, *Asterias amurensis* starfish has been identified as a Ca-rich carbonate material, with XRD analysis confirming calcite as the major mineral phase in natural starfish-derived material (Park et al., 2023). However, the CaCO_3_ content of the SF sample used in this study was not directly quantified, which represents a limitation in accurately defining the inorganic composition of SF and calculating its TBMP. Therefore, future studies should directly quantify the CaCO_3_ content of SF to reduce uncertainty in TBMP estimation. TBMP decreased from 826 to 671 mL CH_4_/g VS as the assumed CaCO_3_ content increased from 50 to 80% FS, consistent with reduced organic carbon availability.

**TABLE 2:** Elemental composition (measured and calculated) and TBMP of dried SF under different assumptions of CaCO_3_ content.

Assumed CaCO_3_ content (% of FS)	Measured				Calculated		
	C (%)	H (%)	N (%)	S (%)	Organic C (%)	O (%)	TBMP (mL CH_4_/g VS)
0	21.4	1.9	2.0	N.D.	21.4	0.0	1,076
50					16.9	2.3	826
70					15.0	4.2	721
80					14.1	5.1	671
90					13.2	6.0	621
100					12.3	6.9	571

N.D, not detected. All percentages are expressed on a TS basis. O% was calculated as O = VS − (organic C + H + N + S). Organic C was calculated by subtracting inorganic C associated with CaCO_3_ (12.0% of the assumed CaCO_3_ content) from total C.

### 3.2 Methane yield of SF

**FIGURE 1:**
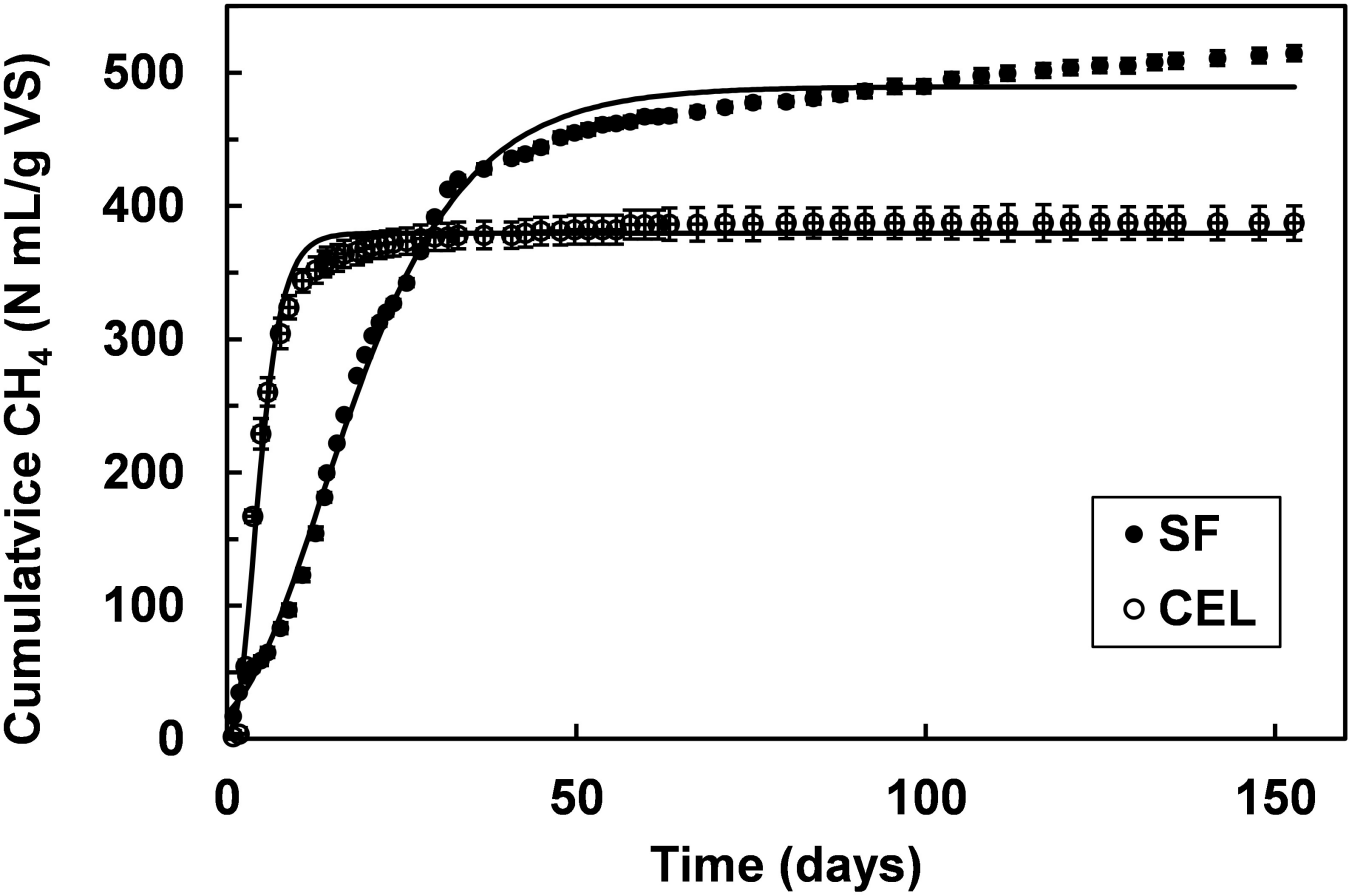
Cumulative methane production from starfish powder SF and CEL with modified Gompertz model fitting. Symbols indicate mean experimental values; solid lines, model predictions.

Cumulative methane production from SF was evaluated in batch tests and compared with the positive control, CEL (Figure 1). At the end of the 152-day operation, methane yields reached 387 mL CH_4_/g VS for CEL and 490 mL CH_4_/g VS for SF. Modified Gompertz model fitting produced similar final yields (380 and 514 mL CH_4_/g VS, respectively; Table 3), with high agreement (*R*^2^ >0.98). Based on Buswell–Boyle calculations, TBMP was 415 mL CH_4_/g VS for CEL and 671–826 mL CH_4_/g VS for SF, assuming 50–80% CaCO_3_ in FS (Table 2). The experimental yields corresponded to 93.3% and 59–73% of TBMP for CEL and SF, respectively. These results suggest that SF can contribute to methane production not only by supplying inorganic CaCO_3_, but also by serving as a substrate containing a biodegradable organic fraction. However, the experimental methane yield of SF was lower than its TBMP, which may reflect the CaCO_3_-rich ossicle structure and differences in the biodegradability of its organic fraction.

**TABLE 3:** Kinetic parameters from the modified Gompertz model for SF and CEL.

Substrate	*B*_0_ (mL CH_4_/g VS)	*R*_*m*_ (mL CH_4_/g VS/day)	λ (day)
SF	514	15.9	2.1
CEL	380	62.8	1.5

*B*_0_, methane production potential; *R*_*m*_, maximum methane production rate; λ, lag phase.

### 3.3 Resumption and multiphase methane production with SF under substrate overload

To investigate the effect of SF as a source of CaCO_3_ and alkalinity supply, batch experiments were conducted under four conditions (Table 4): (i) a baseline with excess starch (30 g VS/L) to induce a pH drop and cessation of methanogenesis (V30_N); (ii) the baseline supplemented with CaCO_3_ at 5, 25, or 50 g/L (Group 1; V30_Ca5, V30_Ca25, V30_Ca50); (iii) the baseline supplemented with the mineral fraction (FS) of SF at CaCO_3_-equivalent doses of 5, 25, or 50 g FS/L, with starch reduced to maintain a total organic matter concentration of 30 g VS/L (Group 2; V30_SF5, V30_SF25, V30_SF50); and (iv) FS of SF at 25 or 50 g/L, corresponding to 7.5 and 15 g VS/L, respectively, with total VS concentrations increased to 37.5 and 45 g VS/L (Group 3; V38_SF25, V45_SF50).

**TABLE 4:** Starch input, supplementation doses of CaCO_3_ and SF, expected methane production calculated from measured yields of the individual substrates (*B*_*ex*_), and final cumulative methane production (*BE*_*end*_).

Trial	Starch	CaCO_3_	SF		*B*_*ex*_	*BE*_*end*_	*BE*_*end*_/*B*_*ex*_
	(g VS/L)	(g/L)	(g VS/L)	(g FS/L)	(mL/bottle)	(mL/bottle)	(%)
V30_N	30.0	0.0	0.0	0.0	780	15.8	2.0
V30_Ca5	30.0	5.0	0.0	0.0	780	14.0	1.8
V30_Ca25	30.0	25.0	0.0	0.0	780	32.8	4.2
V30_Ca50	30.0	50.0	0.0	0.0	780	41.7	5.4
V30_SF5	28.5	0.0	1.5	5.0	769	37.3	4.9
V30_SF25	22.5	0.0	7.5	25.0	739	461	62.4
V30_SF50	15.0	0.0	15.0	50.0	699	546	78.1
V38_SF25	30.0	0.0	7.5	25.0	934	562	60.2
V45_SF50	30.0	0.0	15.0	50.0	1,089	649	59.6

Starch input is expressed in g VS/L, and SF input is expressed as both fixed solids and volatile solids. *B*_*ex*_ = (starch VS × measured CH_4_ yield of starch) + (SF VS × measured CH_4_ yield of SF). *BE*_*end*_ = experimental cumulative methane production at the end of the experiment. *BE*_*end*_/*B*_*ex*_ = percentage of *B*_*ex*_ achieved at the end of the experiment.

**FIGURE 2:**
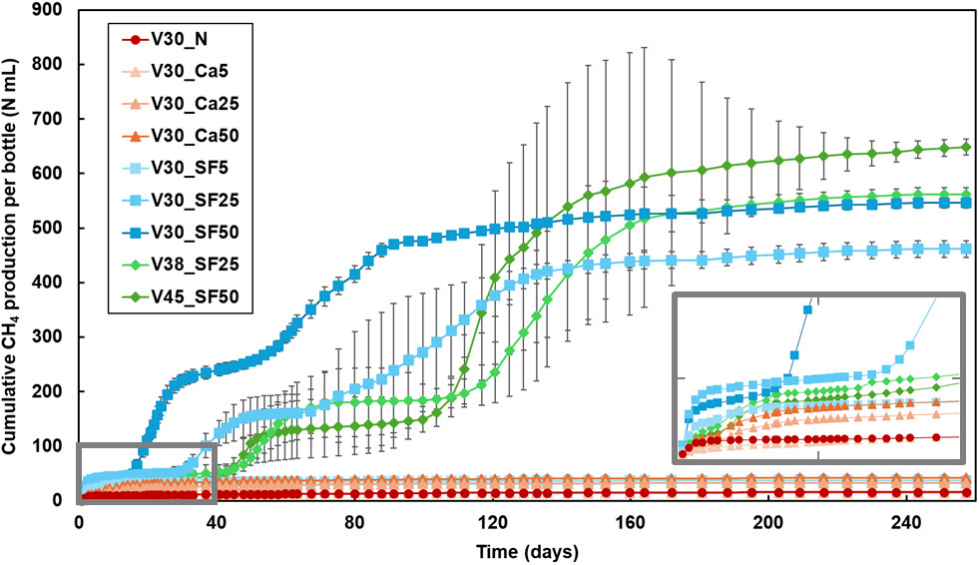
Cumulative methane production from baseline and additive-supplemented treatments during batch digestion. VXX_Y, where “XX” indicates the total VS concentration (g VS/L) and “Y” denotes the additive and its dose: N, no additive; Ca5, CaCO_3_ at 5 g/L; Ca25, CaCO_3_ at 25 g/L; Ca50, CaCO_3_ at 50 g/L; SF5, SF fixed solids (FS) at 5 g/L (CaCO_3_-equivalent); SF25, SF FS at 25 g/L; SF50, SF FS at 50 g/L. V38 and V45 indicate treatments in which SF25 and SF50 were applied without reducing starch, resulting in total VS concentrations of 37.5 and 45 g VS/L, respectively. The gray-bordered inset shows an enlargement of the region from 0 to 40 days (*x*-axis) and 0 to 100 mL (*y*-axis).

Biogas volume and methane content were monitored throughout the 257-day digestion period. As shown in Figure 2 and Table 4, the baseline condition (excess starch only) exhibited minimal methane production, with CH_4_ content remaining in the 20% range and cumulative volume plateauing at <2% of the expected methane production (B_ex_) by day 40. CaCO_3_ supplementation (Group 1) produced only a transient increase in CH_4_ content (up to ∼25%), with final cumulative methane production (*BE*_*end*_) remaining low—ranging from 14.0 to 41.7 mL/bottle, which corresponds to only 1.8–5.4% of *B*_*ex*_, calculated using the measured methane yield of starch as determined in this study (433.4 mL CH_4_/g VS). This low recovery can be explained by the limited role of carbonate-based buffering under acidified conditions. When the pH decreases below approximately 6.3–6.5, bicarbonate shifts toward dissolved CO_2_, which can be readily lost from the liquid phase in open or intermittently depressurized batch systems. Therefore, once acidification had already developed, CaCO_3_ addition alone, even at high doses, was insufficient to sustain buffering capacity or restore methanogenesis.

In contrast, bottles supplemented with the mineral fraction (FS) of SF (Groups 2 and 3) demonstrated significantly enhanced methane production. At 25 or 50 g FS/L, all bottles resumed methanogenesis after an initial stagnation phase and exhibited a distinct triphasic accumulation pattern, marked by stepwise increases in gas volume. CH_4_ content also steadily increased to 50–70% and was sustained thereafter.

SF-supplemented treatments resulted in *BE*_*end*_ values of 461–649 mL/bottle, corresponding to 60–78% of *B*_*ex*_ (Table 4). Moreover, higher total VS loadings further improved absolute methane yields, with the V45_SF50 condition (45 g VS/L total; 15 g VS from SF) showing the highest *B*_*ex*_ and *BE*_*end*_ values (1,089 and 649 mL/bottle, respectively). This contrast suggests that the effect of SF cannot be explained solely by its CaCO_3_ content. Unlike pure CaCO_3_, the SF used in this study was a complex biogenic material containing not only fixed solids but also 23.1% VS; therefore, SF addition should not be interpreted as a direct chemical comparison with freely dispersed CaCO_3_ supplementation. Thus, the difference between CaCO_3_-only and SF-amended treatments is better interpreted as a process-level comparison between a pure CaCO_3_ amendment and an SF additive that combines CaCO_3_, organic matter, minerals, and structural properties. Although their composition and structure are not identical to SF, recent AD studies using crawfish shell waste and chitin-containing shell waste also suggest that biogenic organic–inorganic shell materials can enhance methane production or co-digestion performance through combined effects involving organic substrate supply, CaCO_3_-associated buffering, trace elements, reduced lag time, and methanogenic archaeal colonization (Luo et al., 2023; Xu et al., 2024). At the initial stage of the experiment, freely dispersed CaCO_3_ powder and structurally embedded CaCO_3_ in SF likely differed in particle size, surface exposure, and dissolution behavior. Pure CaCO_3_ could provide more immediate contact with the bulk liquid and thus faster initial neutralization; however, under acidified and intermittently depressurized batch conditions, bicarbonate generated from carbonate dissolution may have shifted toward dissolved CO_2_ and been lost from the liquid phase during gas release. In contrast, CaCO_3_ embedded in the larger and heterogeneous ossicle-derived matrix of SF may have dissolved more gradually during long-term digestion, allowing sustained CaCO_3_ availability and structural support. Therefore, the observed difference between CaCO_3_-only and SF-amended treatments should be interpreted not only as a difference in chemical buffering, but also as a consequence of physical form, dissolution behavior, and structural support. The biodegradable organic fraction of SF may have been slowly degraded during long-term operation, supporting microbial survival and gradual methanogenic recovery. Therefore, the recovery observed in SF-amended treatments should be interpreted as the combined effect of SF-derived biodegradable organic matter, CaCO_3_-rich fixed solids, and porous ossicle structures, rather than as the sole effect of CaCO_3_ supplementation. However, because this study did not include additional controls such as calcined or organic-depleted starfish skeletons, the individual contributions of SF-derived organic matter, biogenic CaCO_3_, and physical structure could not be fully separated. Future studies should include such controls to distinguish the respective contributions of these components to methanogenic recovery. In addition, although end-point alkalinity was additionally measured and added to Supplementary material, bicarbonate concentration, carbonate speciation, time-resolved alkalinity changes, and carbon mass balance were not determined in this study. Therefore, the contribution of carbonate buffering to methane recovery could not be quantitatively separated from the effects of SF-derived organic matter, structural support, and microbial restructuring. Therefore, future studies should also include alkalinity monitoring and carbon balance analysis to verify the proposed buffering mechanism.

**FIGURE 3:**
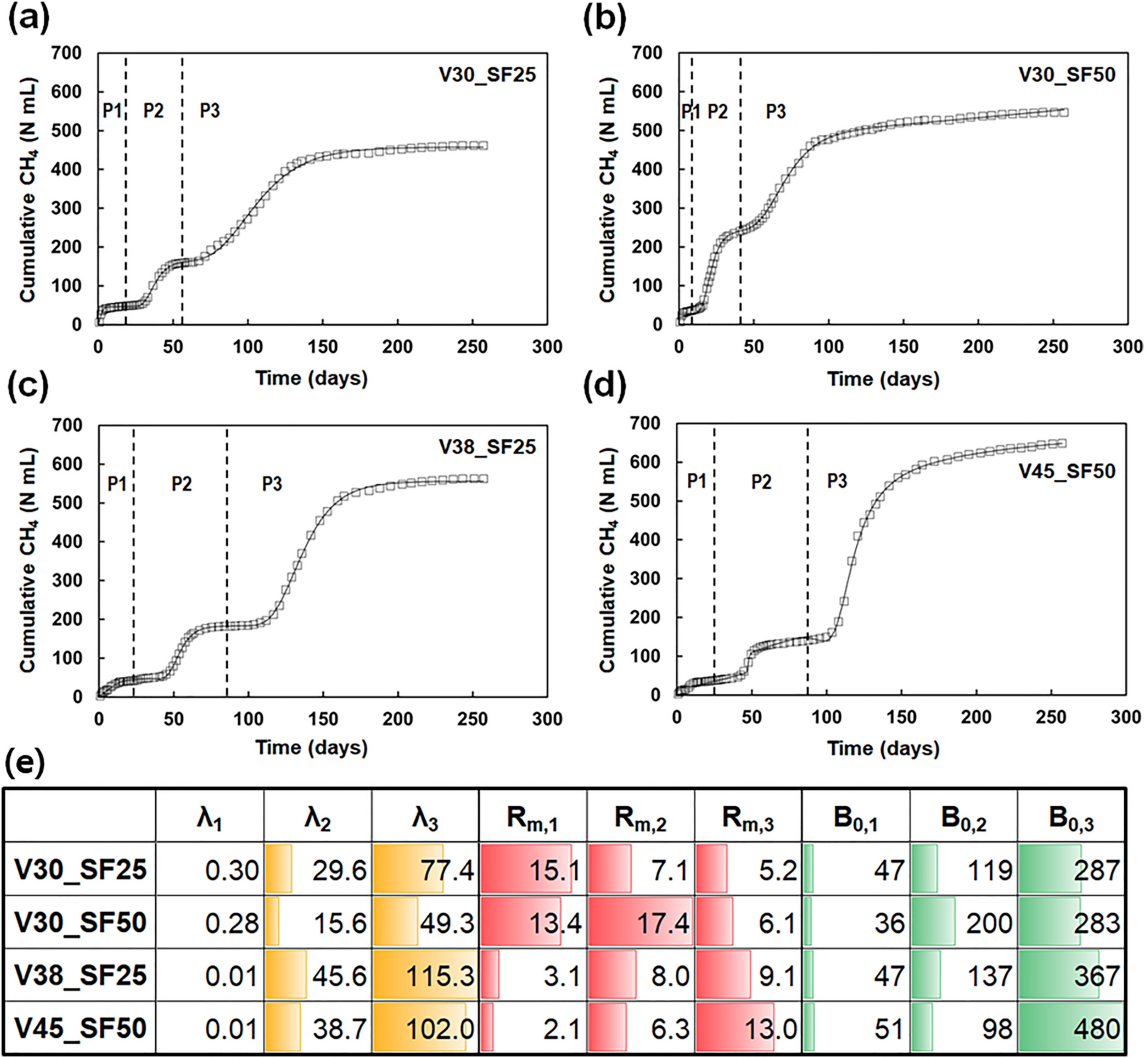
Cumulative methane production and tri-Gompertz model fits for SF-supplemented conditions that resumed methane production: **(a)** V30_SF25, **(b)** V30_SF50, **(c)** V38_SF25, and **(d)** V45_SF50; and **(e)** kinetic parameters estimated from the tri-Gompertz model for each phase under the SF-supplemented conditions. Symbols represent mean experimental data, and solid lines represent model predictions. Phase boundaries (P1–P3) were defined as described in Section 2.5 (Modeling) and are shown as dashed lines. *λ_*i*_* denotes the absolute lag time of phase *i* (days) from day 0 (start of batch), *R_*m*,i_* is the maximum methane production rate of phase *i* (mL CH_4_/day), and *B_0,i_* is the cumulative methane production from phase *i* (mL CH_4_/bottle).

To quantitatively describe the multiphase methane production behavior of SF-treated reactors, cumulative methane production profiles were fitted using a tri-Gompertz model (Figure 3). Similar multiphase gas production behavior has been described in batch anaerobic digestion using two-phase or three-phase kinetic models, particularly when substrate degradation involves fractions with different degradation rates or diauxic production patterns (Gomes et al., 2021; Gouveia et al., 2022). Excellent fits were obtained for all conditions (*R*^2^ >0.99), and the resulting kinetic parameters—lag time (*λ_*i*_*), maximum methane production rate (*R_*m*,i_*), and cumulative methane production (*B*_0,i_), where *i* denotes the phase number—are summarized in Figure 3e. The relatively large error bars observed in the cumulative methane production profiles likely reflected bottle-to-bottle differences in the timing of methanogenic recovery under intentionally induced severe organic overload, rather than a lack of reproducibility in the recovery response. In this study, organic overload was deliberately imposed to induce acidification and to compare the recovery capacity of CaCO_3_ and SF. Under this threshold-like inhibitory condition, small differences among replicate bottles in local buffering, residual methanogenic activity, VFA conversion, and SF particle-size composition could lead to noticeable differences in the onset and progression of methane recovery. Although the SF powder was thoroughly mixed before addition, its particle-size distribution was heterogeneous, with 50.2% of particles smaller than 0.15 mm and approximately 15% larger than 0.85 mm (Table 1). Therefore, slight differences in the particle-size composition introduced into each bottle may have affected CaCO_3_ dissolution, microbial attachment, and the timing of methanogenic recovery. To verify that this variability did not undermine the reproducibility of the triphasic methane production pattern, the tri-Gompertz model was additionally fitted to each replicate bottle. All replicate-level fittings showed high goodness of fit, with *R*^2^ values of 0.997–1.000 and RMSE values of 3.02–9.40 mL/bottle. Although the lag times for Phase II and Phase III varied among replicate bottles, the triphasic methane production pattern was consistently reproduced across all replicates. These results indicate that the error bars in the cumulative methane production profiles mainly represent differences in recovery timing rather than inconsistency in SF-assisted methanogenic recovery.

Based on the tri-Gompertz model fitting, methane production in SF-treated reactors was resolved into three distinct phases. Following an initial plateau (Phase I), methane resumption occurred after a prolonged second lag phase (*λ_2_* = 16–46 d; Phase II), followed by a third lag phase (*λ_3_* = 49–115 d; Phase III). The maximum methane production rates during Phase II and Phase III (*R*_*m,2*_ and *R*_*m,3*_) increased in parallel with the cumulative methane production achieved in the corresponding phases (*B*_0,2_ and *B*_0,3_).

In Phase I, the lag time (*λ_1_*) was negligible across all treatments (0.01–0.30 d), and the maximum methane production rate (*R*_*m,1*_) was at least fourfold higher in Group 2 (low starch, 15–22.5 g VS/L) than in Group 3 (30 g VS/L starch). Despite this, Phase I was short-lived, with cumulative methane production (*B*_0,1_) accounting for only 3.3–7.3% of the total expected methane yield (*B*_*ex*_).

The onset of Phase II (*λ_2_*) varied with starch content and SF loading. When starch content was equal, higher SF input was associated with shorter *λ_2_* values. In Phase II, V30_SF50 exhibited the highest *R*_*m,2*_ and *B*_0,2_ values, which were 2.2–2.8 and 1.5–2.0 times higher than those of the other treatments, respectively.

In Phase III, V45_SF50 showed the highest *R*_*m,3*_ and *B*_0,3_ values, exceeding those of the other treatments by 1.4–2.5 and 1.3–1.7 times, respectively. Collectively, these results demonstrate clear phase-dependent trends, with lower starch loadings associated with shorter second lag times (*λ_2_*) and higher total VS loadings associated with greater cumulative methane production during Phase III (*B*_0,3_). This triphasic behavior indicates that recovery in SF-amended treatments was not a single immediate response but occurred stepwise. Phase I likely reflected initial methanogenesis before severe acidification or degradation of readily available substrates. The prolonged delay before Phase II indicates that bulk conditions remained unfavorable for methanogenesis due to low pH and accumulated VFAs. Such lag periods can be interpreted as the adaptation time required for methanogens to overcome inhibitory conditions, as previously reported for methanogenesis inhibited by ammonia and propionate (Shin et al., 2019). Methanogenic resumption during this phase may have occurred locally around SF particles, where partially buffered microenvironments may have supported limited methanogenic activity. In Phase III, VFA removal and pH recovery likely allowed methanogenesis to expand more broadly in the bulk phase, resulting in greater methane production under higher total VS loading.

### 3.4 End-point pH and VFA concentrations

**FIGURE 4:**
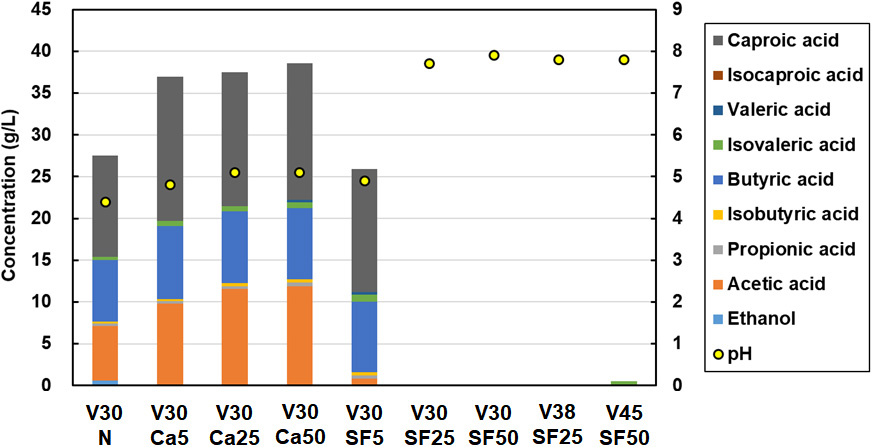
Final pH and total VFA concentrations at the end of the batch experiment for all treatments. Bars represent total VFA concentrations (left *y*-axis), and symbols represent final pH values (right *y*-axis). Treatments with final pH ≥7.5 generally showed low VFA accumulation, whereas those with final pH ≤5.1 exhibited high VFA concentrations.

Final pH and VFA concentrations were measured immediately after the experiment (Figure 4). In CaCO_3_-supplemented conditions, the final pH remained ≤5.1, and high VFA concentrations (>25 g/L), dominated by acetic, butyric, and caproic acids, were detected. This accumulation of VFAs is consistent with the low pH and lack of methanogenic activity. The dominance of caproic acid under starch overloading has been reported under methanogenesis-inhibited conditions and is commonly associated with chain-elongation metabolism (Grootscholten et al., 2013; Nzeteu et al., 2018). These end-point pH and VFA results are consistent with the methane recovery patterns observed above. In the CaCO_3_-amended treatments, VFAs remained accumulated and the pH stayed low, indicating persistent acidification and limited methanogenic activity. In contrast, SF-supplemented conditions—except for the lowest SF input, V30_SF5 (pH 4.88)—resumed methanogenesis, which led to the removal of accumulated VFAs (<0.6 g/L) and a corresponding increase in pH to slightly alkaline levels (7.5–7.9). These results indicate that in SF-supplemented conditions, the resumption of methanogenesis facilitated VFA removal, which in turn led to pH recovery to levels favorable for methanogenesis (7.5–7.9). Additional end-point alkalinity measurements further supported this pattern (Supplementary Table 3). The SF-amended treatments that resumed methanogenesis retained relatively high alkalinity, especially V30_SF50, V38_SF25, and V45_SF50, which showed alkalinity values of 1,621, 1,719, and 4,410 mg CaCO_3_/L, respectively, when titrated to pH 4.3. In contrast, the starch-only and low-dose CaCO_3_-amended treatments showed much lower alkalinity, consistent with persistent acidification and VFA accumulation. However, because alkalinity was measured only at the end point, these data do not resolve the temporal dynamics of bicarbonate buffering or carbon redistribution during recovery.

### 3.5 FE-SEM observations of SF before and after anaerobic digestion

**FIGURE 5:**
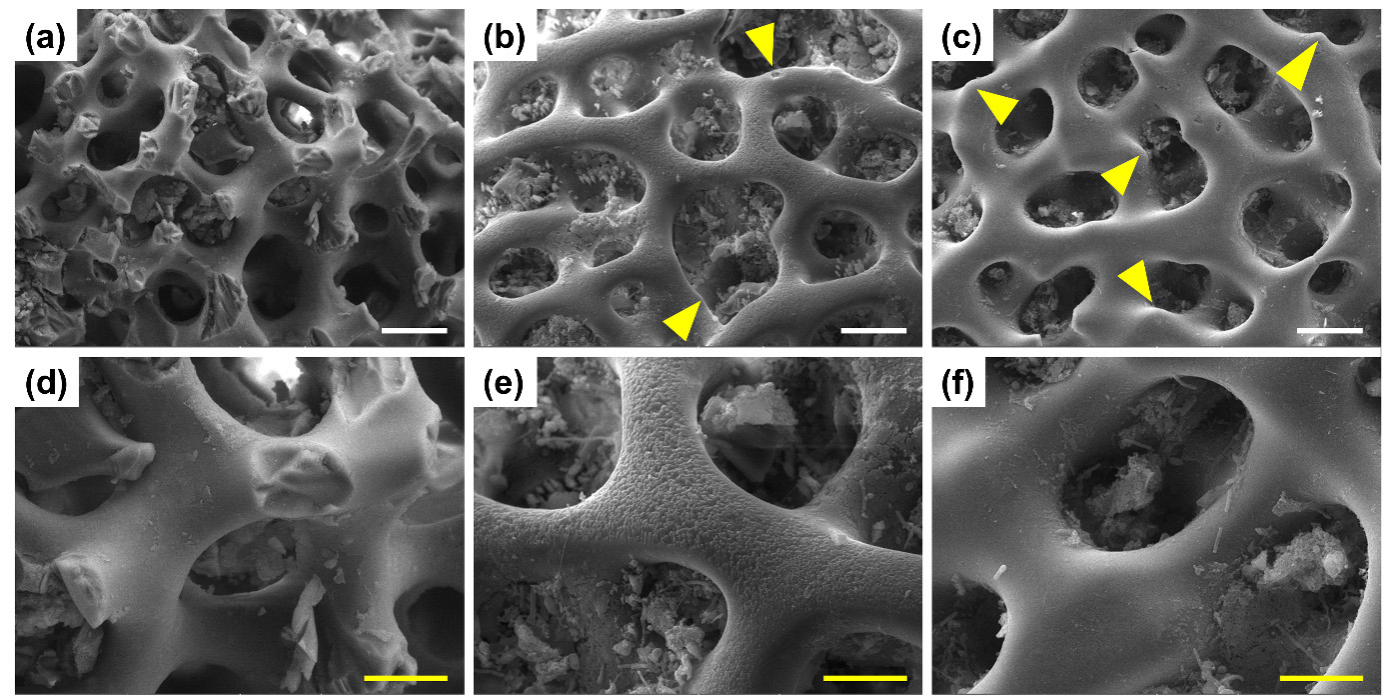
FE-SEM images of SF before and after anaerobic digestion: **(a,d)** raw SF; **(b,e)** V30_SF50; **(c,f)** V45_SF50. White bars = 20 μm; yellow bars = 10 μm. Yellow arrowheads highlight regions where sharp, spiky features observed in raw SF appear to have transitioned into smoother and more rounded edges after anaerobic digestion.

FE-SEM imaging revealed distinct structural changes in SF before and after digestion (Figure 5). Raw SF (Figures 5a,d) showed ossicle-like microscale pores, with sharp, spiky surfaces and pores often occluded by presumed CaCO_3_ crystals formed during grinding. After digestion (V30_SF50: Figures 5b,e; V45_SF50: Figures 5c,f), surfaces became smoother and rounded, with eroded pore edges and enlarged or interconnected openings, likely from CaCO_3_ dissolution in the skeletal framework. Microbial cells and organic debris were frequently observed inside the pores. These FE-SEM observations provide structural support for the different responses observed between SF and freely dispersed CaCO_3_, although both materials contain CaCO_3_ as a major inorganic component. The net-like ossicle-derived microstructure of SF may have provided partially confined porous regions where CaCO_3_ could be retained and gradually dissolved. These microporous structures are consistent with the possibility that SF particles provided partially protected microenvironments where carbonate dissolution and microbial attachment could occur. However, local CO_2_ retention, bicarbonate buffering, and microscale pH were not directly measured, which represents a limitation in directly validating the proposed microenvironment-based recovery mechanism. As a result, microenvironments favorable for partial methanogenic activity may have formed on or within SF particles, even when the bulk conditions were unfavorable for methanogenesis. This behavior contrasts with freely dispersed CaCO_3_ particles, for which buffering capacity may be rapidly diminished under acidic conditions through CO_2_ loss.

### 3.6 End-point microbial community composition

**FIGURE 6:**
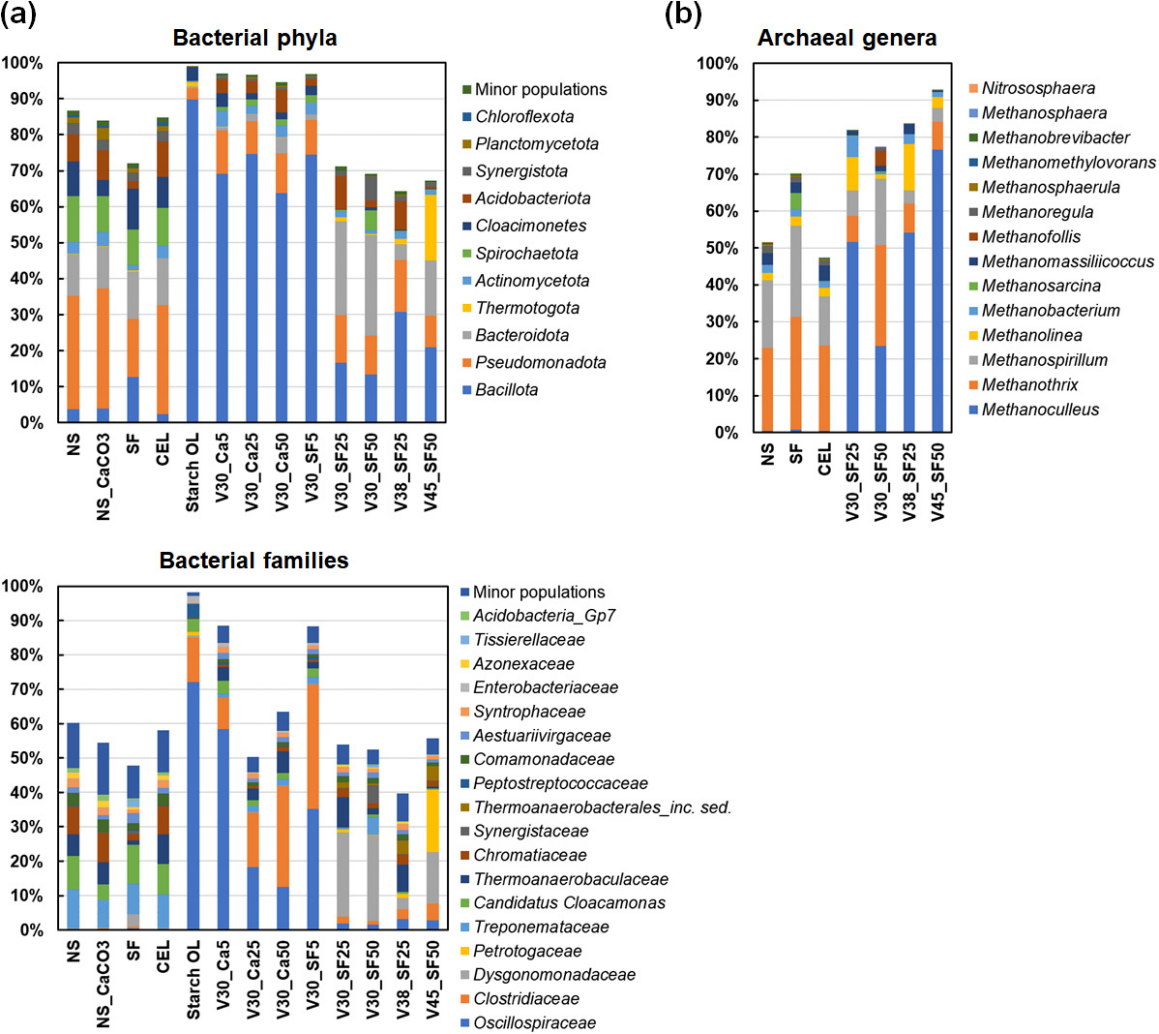
End-point microbial communities from 16S rRNA gene amplicon sequencing: **(a)** bacterial phyla (top) and families (bottom); **(b)** archaeal genera. Archaeal data are shown only for samples without methanogenic failure due to acidification. Minor phyla and families denote taxa with maximum relative abundance <1.0% and <1.5%, respectively. : NS, no substrate; NS_CaCO_3_, no substrate with CaCO_3_ only; SF, starfish as substrate (methane yield test, see Section 3.2); CEL, cellulose as substrate (see section 3.2); starch OL, starch overload at 30 g VS/L. “inc. sed.” denotes *incertae sedis.*

End-point microbial community compositions differed among the control group (NS, NS_CaCO_3_, SF, and CEL), the starch-overloaded CaCO_3_-amended group (V30_Ca), and the starch-overloaded SF-amended group (V30_SF, V38_SF, and V45_SF) (Figure 6 and Supplementary Table 2). Archaeal community data are presented only for samples without methanogenic failure due to acidification.

In the control group, bacterial communities were composed primarily of *Pseudomonadota*, *Bacteroidota*, *Spirochaetota*, and *Cloacimonetes*, while *Bacillota* accounted for < 15% of total sequences. SF and CEL controls showed broadly comparable phylum-level profiles, although SF samples contained a higher proportion of unclassified bacteria.

Under starch overloading, bacterial community structures diverged markedly depending on the amendment. In the acidified CaCO_3_-amended samples (V30_Ca), *Bacillota* strongly dominated (63.8–74.7%), accompanied by pronounced reductions in other major phyla. At the family level, *Oscillospiraceae* and *Clostridiaceae* were the main classified groups. In contrast, SF-amended starch-overloaded samples (V30_SF, V38_SF, and V45_SF) showed a marked decrease in *Bacillota* with increasing SF addition, whereas higher VS loading was associated with a partial increase in *Bacillota*, accompanied by increased contributions from *Bacteroidota* and *Pseudomonadota*. At the family level, *Dysgonomonadaceae* became a prominent group, whereas families abundant in the control group, such as *Treponemataceae* and *Candidatus Cloacamonas*, declined under SF-amended overload conditions. Members of the family *Dysgonomonadaceae* encode diverse glycosyl-hydrolases organized in polysaccharide utilization loci, supporting fermentative degradation of complex carbohydrates and amino acids in AD systems (Maus et al., 2020). In addition, members of *Dysgonomonas* have been reported to hydrolyze starch and ferment glucose with acid production, further supporting their possible role in carbohydrate utilization and fermentative metabolism (Lawson et al., 2002). Therefore, the increase in *Dysgonomonadaceae* should not be interpreted solely as a response to fermentable organic substrates released from SF. This may also suggest the persistence of fermentative populations capable of utilizing complex organic substrates under prolonged acidification and methanogenic inhibition, potentially supporting substrate conversion and subsequent VFA turnover associated with methanogenic recovery.

Archaeal communities in the control group were dominated by *Methanothrix* (22.5–30.6%) and *Methanospirillum* (13.2–24.6%), while *Methanoculleus* accounted for < 1% of total archaeal sequences. In SF-amended starch-overloaded samples, a pronounced shift in archaeal community composition was observed. *Methanoculleus* became dominant in V30_SF25 (51.6%) and at higher total VS loadings (V38_SF25 and V45_SF50), whereas V30_SF50 exhibited co-dominance of *Methanothrix* (27.4%) and *Methanoculleus* (23.4%). Across SF-amended samples, the proportion of unclassified archaeal sequences decreased with increasing SF addition and VS loading, reflecting the emergence of a more clearly defined methanogenic community structure. These community-level shifts are consistent with the observed methane production patterns. The reduced dominance of *Bacillota* in SF-amended conditions may reflect partial alleviation of acidification stress and recovery of functional diversity within the fermentative bacterial community. At the same time, the increased contribution of *Bacteroidota*, which often include hydrolytic and fermentative taxa, may have contributed to the stepwise VFA consumption and triphasic methane recovery observed in SF-amended reactors (Hu et al., 2024). At the archaeal level, methane recovery was associated with a shift toward hydrogenotrophic methanogenesis. *Methanoculleus* dominated under medium and high VS loading conditions, whereas co-dominance with *Methanothrix* was observed under specific SF-amended conditions. This pattern is consistent with previous organic-overload studies in which *Methanoculleus*- and *Methanospirillum*-related hydrogenotrophic methanogens became detectable or more dominant under increasing VFA concentrations (Lerm et al., 2012). This shift toward hydrogenotrophic methanogenesis is consistent with previous knowledge regarding differences in pH tolerance between aceticlastic and hydrogenotrophic methanogens. Under acidic conditions, aceticlastic methanogens such as *Methanothrix* can be selectively inhibited, whereas hydrogenotrophic methanogens generally show higher acid tolerance and can persist over lower pH ranges (Wang et al., 2020). Therefore, the dominance of *Methanoculleus* under SF-amended overload conditions may reflect the selective enrichment of acid-tolerant hydrogenotrophic methanogens within partially buffered environments provided by SF. Taken together, these microbial results suggest that SF addition did not simply restore the original non-acidified community structure, but may have promoted the formation of an alternative methanogenic community structure compatible with sustained methane production under severe organic overload. This microbial restructuring, together with the localized buffering and structural support provided by SF, likely contributed to the long-term recovery observed in SF-amended reactors. However, because the microbial analysis was based on end-point 16S rRNA gene amplicon sequencing, the inferred metabolic roles of bacterial and archaeal taxa were not functionally validated. Metagenomic, transcriptomic, or activity-based analyses would be required to directly confirm the metabolic pathways responsible for SF-assisted recovery.

Another possible but unverified contribution of SF is the supply of trace metals such as Fe, Ni, Co, and Zn, which are important cofactors for enzymes involved in syntrophic metabolism and methanogenesis. Previous analyses of starfish biomass have detected major and trace elements, including Fe, Co, Ni, and Zn, in *Asterias rubens* and *Marthasterias glacialis* (Turull et al., 2025). Although trace metal concentrations were not measured in this study, their potential release from SF could have supported archaeal activity and contributed to methanogenic recovery. Therefore, future studies should quantify trace metal release from SF and evaluate its contribution separately from alkalinity release, organic matter degradation, and structural effects. Overall, these findings suggest that SF-assisted recovery under severe organic overload was not driven by a single factor, but by the combined effects of biodegradable organic matter, CaCO_3_-associated buffering, porous structural support, microbial community restructuring, and possibly trace-metal-related support. Although some mechanisms remain inferential, the integrated methane production, pH/VFA, FE-SEM, and microbial community results indicate that SF can function as a slow-acting stabilizing additive for long-term methanogenic recovery rather than as a rapid-response pH-control reagent, particularly given the static batch configuration and prolonged lag phases observed in this study.

### 3.7 Influence of SF on practical application in overloaded AD systems

Considering the prolonged recovery lag observed under the intentionally severe starch overloading condition of 30 g VS/L, SF should not be regarded as a rapid-response additive for immediate process recovery. Instead, SF is better positioned as a methane-producing co-substrate and slow-acting pH-stabilizing amendment for overloaded AD systems where long-term recovery and process resilience are prioritized over rapid intervention.

This application may be particularly relevant when methanogenesis is inhibited by organic overloading, the use of strong alkalis such as NaOH is impractical, and additional Ca^2+^ input is acceptable despite its potential to promote precipitation or scaling (Zhang et al., 2015). Potential application scenarios include one-time treatment of large biomass loads, long-term anaerobic storage or landfill-type methane generation, and small or remote digesters with limited process control. In systems where digestate is applied to land, additional Ca^2+^ may also be beneficial for acidic soils.

From a practical perspective, pulverization can enhance contact between SF particles and the surrounding liquid phase, potentially improving carbonate dissolution and microbial colonization. However, when processing capacity is limited, the use of fresh or coarsely crushed starfish may also be considered as a lower-processing alternative. Overall, SF should be interpreted as a niche, slow-acting stabilizer that supports long-term methanogenic recovery and resource valorization, rather than as an emergency buffering agent for rapid process correction.

## 4 Conclusion

This study demonstrated that SF functions as a multifunctional additive in anaerobic digestion, simultaneously providing organic substrate, alkalinity, and microbial support. As a sole substrate, SF achieved methane yields of 490 mL CH_4_/g VS, corresponding to 59–73% of its theoretical potential. Under starch-induced overload, CaCO_3_ supplementation alone failed to restore methanogenesis, whereas SF addition enabled recovery with triphasic methane production, reaching 60–78% of the expected yield. Microscopic and microbial analyses indicated that SF promoted recovery through progressive CaCO_3_ availability associated with its ossicle microstructure, potential formation of microniches for microbial colonization and enrichment of hydrogenotrophic methanogens accompanied by restructuring of fermentative bacterial communities. These combined physicochemical and microbial effects provided slow but sustained stabilization of overloaded AD. Overall, SF represents a niche, slow-acting alkalinity source that can enhance long-term stability in overloaded digesters, particularly where rapid intervention is less critical, but resilience and resource valorization are prioritized.

## Data Availability

The raw 16S rRNA gene amplicon sequencing data generated in this study have been publicly deposited in the NCBI Sequence Read Archive under BioProject accession number PRJNA1455137.
