# Supplementary material for: Methanogenic recovery under organic overload using starfish-derived powder
Source: Front Microbiol. 2026 Jun 17;17:1853600. doi: 10.3389/fmicb.2026.1853600 (PMC13319069; doi:10.3389/fmicb.2026.1853600)
Supplement: Supplementary file 1 [file Data_Sheet_1.docx]

Table S1. Tri-Gompertz kinetic parameters obtained from individual replicate and mean cumulative methane production curve fittings

| **Bottle** | **λ_1_** | **λ_2_** | **λ_3_** | **R_m,1_** | **R_m,2_** | **R_m,3_** | **B_0,1_** | **B_0,2_** | **B_0,3_** | **B_0,total_** | **RMSE** | **R^2^** |
| --- | --- | --- | --- | --- | --- | --- | --- | --- | --- | --- | --- | --- |
| **V30_SF25_A** | 0.19 | 30.96 | 91.85 | 11.18 | 6.42 | 7.97 | 40.6 | 95.6 | 329.6 | 465.8 | 4.11 | 0.999 |
| **V30_SF25_B** | 0.29 | 29.42 | 89.10 | 16.08 | 6.47 | 6.77 | 50.9 | 96.3 | 289.6 | 436.8 | 5.04 | 0.999 |
| **V30_SF25_C** | 0.35 | 30.18 | 60.48 | 17.62 | 11.35 | 6.15 | 49.4 | 139.4 | 262.4 | 451.1 | 8.73 | 0.997 |
| **V30_SF50_A** | 0.13 | 15.21 | 44.90 | 12.10 | 16.68 | 5.90 | 40.3 | 178.7 | 312.5 | 531.5 | 7.65 | 0.998 |
| **V30_SF50_B** | 0.00 | 15.94 | 46.73 | 10.37 | 20.10 | 5.43 | 41.0 | 200.9 | 298.1 | 540.0 | 8.74 | 0.998 |
| **V30_SF50_C** | 0.58 | 16.19 | 49.22 | 17.96 | 17.57 | 5.50 | 30.6 | 199.7 | 292.6 | 522.9 | 7.05 | 0.999 |
| **V38_SF25_A** | 0.00 | 48.31 | 110.88 | 4.04 | 9.43 | 10.70 | 55.5 | 142.2 | 363.8 | 561.5 | 7.02 | 0.999 |
| **V38_SF25_B** | 0.55 | 43.69 | 125.56 | 2.45 | 7.92 | 10.90 | 38.2 | 130.4 | 374.4 | 543.0 | 4.25 | 1.000 |
| **V38_SF25_C** | 0.00 | 91.33 | 146.04 | 7.47 | 8.41 | 10.73 | 71.0 | 157.0 | 328.1 | 556.1 | 3.63 | 1.000 |
| **V45_SF50_A** | 0.00 | 84.25 | 161.50 | 1.73 | 4.63 | 9.43 | 40.0 | 102.9 | 489.0 | 632.0 | 3.02 | 1.000 |
| **V45_SF50_B** | 0.00 | 43.92 | 104.68 | 0.75 | 10.26 | 16.05 | 200.0 | 63.6 | 412.2 | 675.7 | 8.99 | 0.999 |
| **V45_SF50_C** | 0.33 | 41.58 | 102.89 | 3.43 | 8.61 | 11.75 | 47.4 | 99.4 | 482.4 | 629.3 | 9.40 | 0.998 |
| **V30_SF25** | 0.29 | 29.69 | 76.57 | 15.04 | 7.27 | 5.05 | 46.7 | 117.6 | 293.1 | 457.4 | 4.67 | 0.999 |
| **V30_SF50** | 0.21 | 15.74 | 46.79 | 12.47 | 17.96 | 5.58 | 37.0 | 193.4 | 301.1 | 531.5 | 7.67 | 0.998 |
| **V38_SF25** | 0.00 | 43.62 | 109.40 | 4.55 | 4.50 | 6.49 | 52.8 | 103.3 | 411.1 | 567.3 | 4.97 | 0.999 |
| **V45_SF50** | 0.00 | 43.08 | 88.65 | 2.36 | 7.66 | 5.75 | 39.3 | 51.2 | 550.2 | 640.7 | 15.63 | 0.995 |

λᵢ, Rₘ,ᵢ, and B₀,ᵢ denote the lag time, maximum methane production rate, and methane production potential of phase i, respectively; B₀,total denotes the sum of B₀,₁, B₀,₂, and B₀,₃. Units are days for λᵢ, mL CH₄/day for Rₘ,ᵢ, and mL CH₄/bottle for B₀,ᵢ, B₀,total, and RMSE. Rows labeled with A, B, and C represent parameters obtained by fitting the tri-Gompertz model separately to individual replicate bottles. The last four rows represent parameters obtained by fitting the model to the mean cumulative methane production curves shown in Figure 3; these values are not averages of the parameters obtained from the individual replicate fittings. R² indicates the coefficient of determination for each fitting.

Table S2. Relative abundance of (a) bacterial phyla, (b) bacterial families, and (c) archaeal genera in the samples.

(a) Bacterial phyla

| **Bacterial phyla** | **NS** | **NS_**  **CaCO_3_** | **SF** | **CEL** | **Starch OL** | **V30_**  **Ca5** | **V30_**  **Ca25** | **V30_**  **Ca50** | **V30_**  **SF5** | **V30_**  **SF25** | **V30_**  **SF50** | **V38_**  **SF25** | **V45_**  **SF50** |
| --- | --- | --- | --- | --- | --- | --- | --- | --- | --- | --- | --- | --- | --- |
| ***Bacillota*** | 3.83% | 3.90% | 12.62% | 2.29% | 89.81% | 69.12% | 74.70% | 63.77% | 74.43% | 16.69% | 13.33% | 30.77% | 20.98% |
| ***Pseudomonadota*** | 31.46% | 33.43% | 16.27% | 30.31% | 3.03% | 12.01% | 9.11% | 11.14% | 9.68% | 13.21% | 10.82% | 14.42% | 8.71% |
| ***Bacteroidota*** | 11.55% | 11.57% | 13.08% | 13.10% | 0.65% | 1.00% | 1.98% | 4.29% | 1.53% | 26.05% | 28.01% | 4.33% | 15.33% |
| ***Thermotogota*** | 0.05% | 0.04% | 0.29% | 0.03% | 1.06% | 0.07% | 0.05% | 0.11% | 0.06% | 1.18% | 0.30% | 1.52% | 18.28% |
| ***Actinomycetota*** | 3.40% | 4.01% | 1.61% | 3.44% | 0.40% | 4.45% | 2.17% | 3.21% | 3.23% | 1.70% | 1.10% | 2.03% | 1.32% |
| ***Spirochaetota*** | 12.60% | 9.99% | 9.86% | 10.46% | 0.02% | 1.12% | 1.82% | 1.78% | 2.17% | 0.38% | 5.50% | 0.25% | 0.24% |
| ***Cloacimonetes*** | 9.64% | 4.42% | 11.33% | 8.75% | 3.75% | 3.75% | 1.67% | 1.85% | 2.45% | 0.19% | 0.72% | 0.35% | 0.09% |
| ***Acidobacteriota*** | 7.69% | 8.39% | 2.06% | 9.77% | 0.20% | 4.03% | 3.48% | 6.38% | 2.01% | 9.06% | 2.02% | 7.99% | 0.67% |
| ***Synergistota*** | 3.02% | 2.92% | 2.42% | 2.81% | 0.05% | 0.61% | 0.75% | 0.74% | 0.61% | 1.14% | 6.47% | 1.34% | 1.00% |
| ***Planctomycetota*** | 1.64% | 3.15% | 1.13% | 1.50% | 0.04% | 0.26% | 0.22% | 0.29% | 0.18% | 0.30% | 0.23% | 0.28% | 0.17% |
| ***Chloroflexota*** | 0.64% | 0.73% | 0.23% | 0.93% | 0.01% | 0.05% | 0.07% | 0.11% | 0.05% | 0.13% | 0.05% | 0.08% | 0.05% |
| **Minor populations (max<1.0%)** | 1.28% | 1.38% | 1.09% | 1.43% | 0.00% | 0.50% | 0.65% | 0.94% | 0.53% | 1.13% | 0.55% | 1.01% | 0.40% |
| **Unclassified** | 13.20% | 16.08% | 28.00% | 15.19% | 0.98% | 3.04% | 3.34% | 5.38% | 3.06% | 28.83% | 30.89% | 35.65% | 32.76% |

(b) Bacterial families

| **Bacterial families** | **NS** | **NS_**  **CaCO_3_** | **SF** | **CEL** | **Starch**  **OL** | **V30_**  **Ca5** | **V30_**  **Ca25** | **V30_**  **Ca50** | **V30_**  **SF5** | **V30_**  **SF25** | **V30_**  **SF50** | **V38_S**  **F25** | **V45_**  **SF50** |
| --- | --- | --- | --- | --- | --- | --- | --- | --- | --- | --- | --- | --- | --- |
| ***Oscillospiraceae*** | 0.24% | 0.35% | 0.46% | 0.24% | 72.20% | 58.42% | 18.23% | 12.56% | 35.30% | 1.81% | 1.55% | 3.22% | 2.88% |
| ***Clostridiaceae*** | 0.06% | 0.06% | 0.53% | 0.02% | 12.93% | 9.22% | 15.99% | 29.23% | 36.03% | 2.01% | 1.10% | 2.91% | 4.86% |
| ***Dysgonomonadaceae*** | 0.21% | 0.17% | 3.68% | 0.28% | 0.59% | 0.08% | 0.10% | 0.26% | 0.18% | 24.49% | 24.85% | 3.08% | 14.87% |
| ***Petrotogaceae*** | 0.01% | 0.01% | 0.00% | 0.00% | 1.05% | 0.00% | 0.00% | 0.01% | 0.00% | 1.05% | 0.13% | 1.39% | 18.22% |
| ***Treponemataceae*** | 11.37% | 8.22% | 8.75% | 9.82% | 0.02% | 0.99% | 1.73% | 1.72% | 2.08% | 0.20% | 5.20% | 0.13% | 0.16% |
| ***Candidatus Cloacamonas*** | 9.64% | 4.42% | 11.33% | 8.75% | 3.75% | 3.75% | 1.67% | 1.85% | 2.45% | 0.19% | 0.72% | 0.35% | 0.09% |
| ***Thermoanaerobaculaceae*** | 6.39% | 6.52% | 1.32% | 8.74% | 0.18% | 3.95% | 3.42% | 6.31% | 1.91% | 8.92% | 1.80% | 7.86% | 0.60% |
| ***Chromatiaceae*** | 7.94% | 8.40% | 1.69% | 8.25% | 0.07% | 0.71% | 0.66% | 1.23% | 0.46% | 2.45% | 1.54% | 2.99% | 1.61% |
| ***Synergistaceae*** | 0.21% | 0.25% | 1.17% | 0.10% | 0.01% | 0.09% | 0.08% | 0.04% | 0.12% | 0.18% | 5.23% | 0.20% | 0.27% |
| ***Thermoanaerobacterales_***  ***inc. sed.*** | 0.00% | 0.00% | 0.00% | 0.00% | 0.03% | 0.00% | 0.00% | 0.00% | 0.00% | 1.62% | 0.42% | 3.92% | 4.06% |
| ***Peptostreptococcaceae*** | 0.20% | 0.04% | 0.15% | 0.03% | 4.02% | 0.60% | 0.03% | 0.13% | 0.76% | 0.11% | 0.08% | 0.20% | 0.08% |
| ***Comamonadaceae*** | 3.67% | 3.79% | 2.04% | 3.55% | 0.05% | 0.95% | 1.00% | 1.27% | 0.97% | 1.72% | 1.53% | 1.64% | 0.97% |
| ***Aestuariivirgaceae*** | 1.52% | 1.17% | 2.91% | 1.54% | 0.09% | 1.85% | 1.16% | 1.45% | 1.33% | 1.16% | 1.60% | 1.25% | 0.90% |
| ***Syntrophaceae*** | 2.61% | 2.26% | 0.93% | 2.24% | 0.17% | 1.77% | 1.34% | 1.37% | 1.20% | 1.59% | 1.15% | 1.81% | 1.06% |
| ***Enterobacteriaceae*** | 0.20% | 0.02% | 0.02% | 0.10% | 1.94% | 0.92% | 0.21% | 0.31% | 0.51% | 0.06% | 0.02% | 0.04% | 0.02% |
| ***Azonexaceae*** | 1.65% | 1.94% | 0.77% | 1.31% | 0.02% | 0.14% | 0.20% | 0.17% | 0.18% | 0.46% | 0.37% | 0.41% | 0.26% |
| ***Tissierellaceae*** | 0.05% | 0.01% | 1.91% | 0.00% | 0.00% | 0.00% | 0.00% | 0.00% | 0.00% | 0.02% | 0.66% | 0.05% | 0.11% |
| ***Acidobacteria_Gp7*** | 1.16% | 1.74% | 0.68% | 0.82% | 0.02% | 0.05% | 0.03% | 0.05% | 0.08% | 0.12% | 0.18% | 0.12% | 0.08% |
| **Minor populations**  **(max<1.5%)** | 0.00% | 0.00% | 0.00% | 0.00% | 0.00% | 0.00% | 0.00% | 0.00% | 0.00% | 0.00% | 0.00% | 0.00% | 0.00% |
| **Unclassified** | 39.75% | 45.49% | 52.25% | 41.95% | 1.69% | 11.42% | 49.63% | 36.55% | 11.59% | 46.09% | 47.49% | 60.23% | 44.27% |

(c) Archaeal genera

| **Archaeal genera** | **NS** | **SF** | **CEL** | **V30_SF25** | **V30_SF50** | **V38_SF25** | **V45_SF50** |
| --- | --- | --- | --- | --- | --- | --- | --- |
| ***Methanoculleus*** | 0.50% | 0.73% | 0.53% | 51.55% | 23.44% | 54.11% | 76.66% |
| ***Methanothrix*** | 22.52% | 30.63% | 23.16% | 7.17% | 27.41% | 7.97% | 7.56% |
| ***Methanospirillum*** | 18.21% | 24.58% | 13.22% | 6.77% | 17.87% | 3.54% | 3.60% |
| ***Methanolinea*** | 1.96% | 2.56% | 2.38% | 9.12% | 1.14% | 12.59% | 3.03% |
| ***Methanobacterium*** | 2.16% | 1.95% | 1.65% | 5.79% | 0.57% | 2.60% | 1.32% |
| ***Methanosarcina*** | 0.06% | 4.47% | 0.05% | 0.05% | 0.31% | 0.08% | 0.00% |
| ***Methanomassiliicoccus*** | 3.27% | 2.86% | 4.25% | 1.15% | 1.51% | 2.58% | 0.44% |
| ***Methanofollis*** | 0.00% | 0.00% | 0.00% | 0.00% | 4.10% | 0.00% | 0.00% |
| ***Methanoregula*** | 1.79% | 1.45% | 1.31% | 0.19% | 0.79% | 0.20% | 0.12% |
| ***Methanosphaerula*** | 0.53% | 0.30% | 0.40% | 0.06% | 0.10% | 0.02% | 0.02% |
| ***Methanomethylovorans*** | 0.09% | 0.04% | 0.05% | 0.00% | 0.00% | 0.00% | 0.00% |
| ***Methanobrevibacter*** | 0.41% | 0.48% | 0.34% | 0.07% | 0.06% | 0.07% | 0.02% |
| ***Methanosphaera*** | 0.00% | 0.03% | 0.01% | 0.00% | 0.01% | 0.00% | 0.00% |
| ***Nitrososphaera*** | 0.02% | 0.00% | 0.00% | 0.00% | 0.00% | 0.00% | 0.00% |
| **Unclassified** | 48.47% | 29.92% | 52.64% | 18.06% | 22.67% | 16.24% | 7.23% |

Table S3. End-point alkalinity of digestates after batch digestion.

| **Treatment** | **Alkalinity to pH 4.3 (as mg CaCO_3_/L)** | **Alkalinity to pH 3.8 (as mg CaCO_3_/L)** |
| --- | --- | --- |
| V30_N | 0 | 267.7 |
| V30_Ca5 | 44.7 | 118.1 |
| V30_Ca25 | 257.2 | 363.6 |
| V30_Ca50 | 769.2 | 880.4 |
| V30_SF5 | 209.3 | 357.3 |
| V30_SF25 | 659.3 | 785.7 |
| V30_SF50 | 1621.2 | 1826.0 |
| V38_SF25 | 1719.0 | 1978.3 |
| V45_SF50 | 4410.3 | 4761.8 |


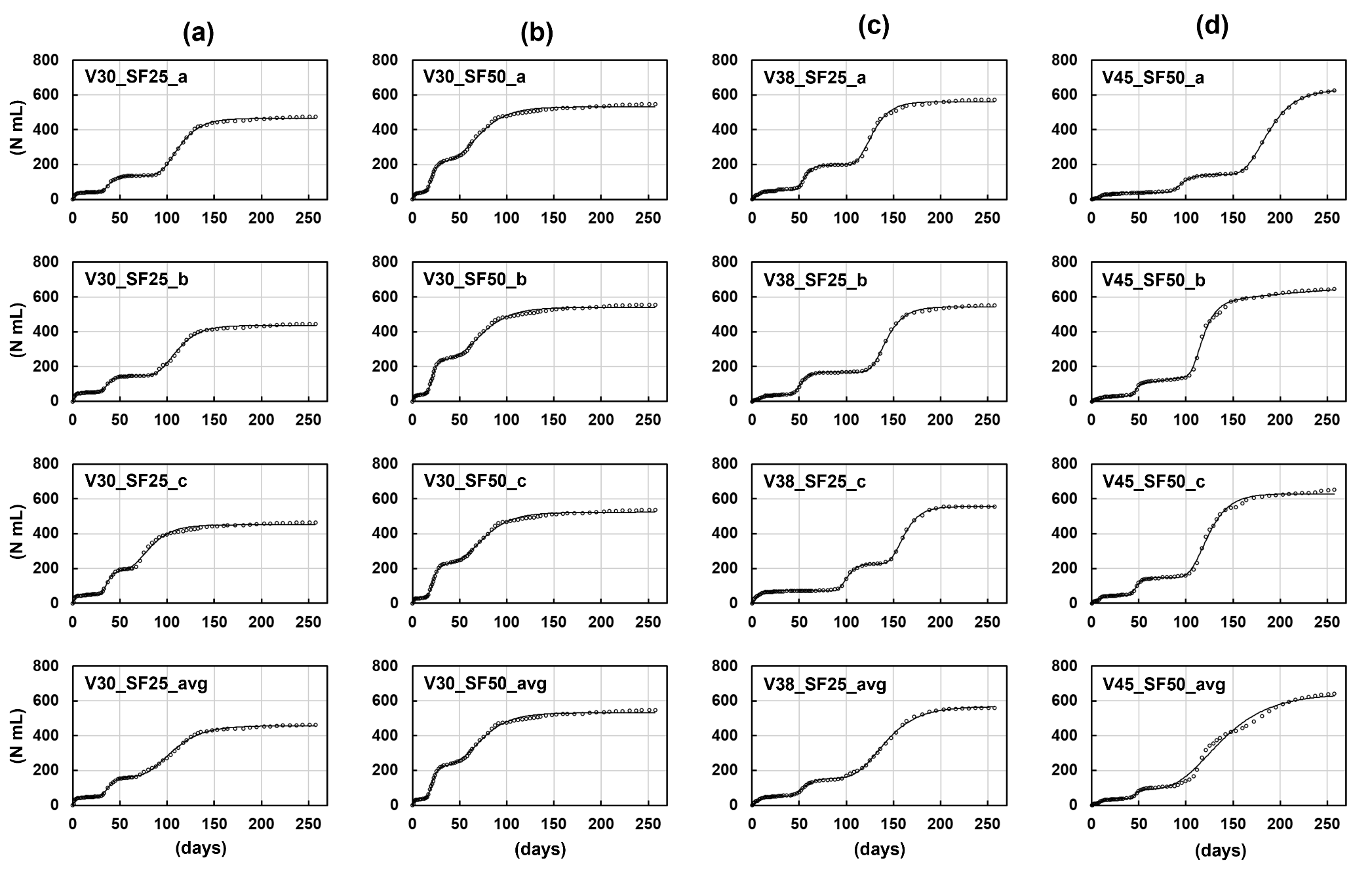


**Figure S1. Tri-Gompertz model fitting of cumulative methane production curves for individual replicates and mean experimental values.** Panels correspond to (a) V30_SF25, (b) V30_SF50, (c) V38_SF25, and (d) V45_SF50. Symbols and solid lines indicate the experimental and fitted values, respectively. The x-axis represents time (days), and the y-axis represents cumulative CH_4_ production (N mL/bottle). The first to third rows show individual replicate fittings, whereas the bottom row shows fittings based on the mean experimental values of the three replicates.
